# Identifying the Effect of COVID-19 Infection in Multiple Myeloma and Diffuse Large B-Cell Lymphoma Patients Using Bioinformatics and System Biology

**DOI:** 10.1155/2022/7017317

**Published:** 2022-11-23

**Authors:** Chengcheng Li, Ying Zhang, Yingying Xiao, Yun Luo

**Affiliations:** ^1^Department of Hematology, The Second Affiliated Hospital of Chongqing Medical University, Chongqing, China; ^2^Institute of Life Science, Chongqing Medical University, Chongqing, China

## Abstract

The severe acute respiratory syndrome coronavirus type 2 (SARS-CoV-2), also referred to as COVID-19, has spread to several countries and caused a serious threat to human health worldwide. Patients with confirmed COVID-19 infection spread the disease rapidly throughout the region. Multiple myeloma (MM) and diffuse large B-cell lymphoma (DLBCL) are risk factors for COVID-19, although the molecular mechanisms underlying the relationship among MM, DLBCL, and COVID-19 have not been elucidated so far. In this context, transcriptome analysis was performed in the present study to identify the shared pathways and molecular indicators of MM, DLBCL, and COVID-19, which benefited the overall understanding of the effect of COVID-19 in patients with MM and DLBCL. Three datasets (GSE16558, GSE56315, and GSE152418) were downloaded from the Gene Expression Omnibus (GEO) and searched for the shared differentially expressed genes (DEGs) in patients with MM and DLBCL who were infected with SARS-CoV-2. The objective was to detect similar pathways and prospective medicines. A total of 29 DEGs that were common across these three datasets were selected. A protein-protein interaction (PPI) network was constructed using data from the STRING database followed by the identification of hub genes. In addition, the association of MM and DLBCL with COVID-19 infection was analyzed through functional analysis using ontologies terms and pathway analysis. Three relationships were observed in the evaluated datasets: transcription factor-gene interactions, protein-drug interactions, and an integrated regulatory network of DEGs and miRNAs with mutual DEGs. The findings of the present study revealed potential pharmaceuticals that could be beneficial in the treatment of COVID-19.

## 1. Introduction

The coronavirus associated with severe acute respiratory syndrome type 2 (SARS-CoV-2) is a single-stranded RNA virus, which is commonly referred to as COVID-19 [1–3]. Numerous studies have reported the risk factors for COVID-19, among which the ones regarded as high-risk factors include cancer, older age, and immunodeficiency [4]. In addition, patients with hematological malignancies (HM), such as multiple myeloma (MM) and diffuse large B-cell lymphoma (DLBCL), present further severe symptoms and a higher fatality rate compared to patients with other kinds of cancers. This is attributed to the greatly reduced immune function in the above two diseases. MM and DLBCL are also reported as high-risk factors for COVID-19 [5–9]. Moreover, recent studies have highlighted that the immune response to COVID-19 mRNA vaccination in MM patients, evaluated based on the neutralizing capability of vaccine-induced antibodies, is significantly compromised in the case of COVID-19 variants [10]. Accordingly, the risk of breakthrough infection with COVID-19 variants was also significantly higher in patients with MM [11].

In the present study, three datasets were analyzed to determine the clinical relationship of COVID-19 with MM and DLBCL. The three datasets, namely, GSE16558, GSE56315, and GSE152418, for COVID-19, MM, and DLBCL, respectively, were downloaded from the Gene Expression Omnibus (GEO) database. First, the differentially expressed gene (DEGs) were identified from these datasets. Next, the common DEGs of the above three disorders were identified. These common DEGs, which were critical for the experiment, were subjected to pathway analysis and enrichment analysis to attain deeper insights into the biological processes associated with the genome-based expression. Afterward, the protein-protein interactions (PPIs) network was constructed based on these common DEGs, and from this network, hub genes were identified. The hub genes would enable the prediction of potential drugs for the diseases. Finally, the miRNA-TF-mRNA network was established. According to the results, recommendations of the potential drugs for treating the three diseases were provided.

## 2. Materials and Methods

### 2.1. Datasets Used

In order to verify the common genetic correlations among COVID-19, MM, and DLBCL, microarray and RNA-seq datasets were downloaded from the GEO database (GEO; https://www.ncbi.nlm.nih.gov/geo/) [12]. The COVID-19 dataset with the accession ID GSE152418 was used as entry into the GEO and subjected to high throughput sequencing Illumina NovaSeq 6000 for RNA-seq analysis [13]. The MM dataset with the GEO accession ID GSE16558 comprised the data of human peripheral blood samples, including sixty bone marrow (BM) samples and five normal controls, obtained using the GPL6244 [HuGene-1_0-st] Affymetrix Human Gene 1.0 ST Array [14]. The inclusion criteria used for sample selection in the case of the MM microarray dataset were as follows: normal bone marrow plasma cell samples from MM patients (≥40); no restrictions on patients' gender, race, treatment response, karyotype, mutation, and pathologic stages. The DLBCL dataset of accession ID GSE56315(GEO) comprised data from 55 patients with DLBCL and 33 normal tonsillar tissue samples [15, 16]. The inclusion and exclusion criteria for this database were obtained from a previous study [15]. The data series was derived using the GPL570 [HG-U133_Plus_2] Affymetrix Human Genome U133 Plus 2.0 Array.

### 2.2. Identification of the DEGs and Shared DEGs among MM, DLBCL, and COVID-19

The Linear Models for Microarray Data (LIMMA) package were employed to identify the significant DEGs from the datasets using the criteria of *P* value < 0.05 and |logFC| ≥ 1.0. The DEGs were identified based on the long-expression values obtained using the LIMMA package with Benjamini-Hochberg's correction to limit the rate of false discovery. Among the identified DEGs, the shared DEGs among MM, DLBCL, and COVID-19 were identified using DESEq2 in the R programming package (v 4.0.2) with different testing options. The shared DEGs common among the datasets GSE16558, GSE56315, and GSE152418 were obtained using a VENN analysis tool named Jvenn [17].

### 2.3. Gene Ontology and Pathway Enrichment Analysis

Gene set enrichment analysis (GSEA) defines the gene expression data and reveals several common biological pathways [18]. EnrichR (http://amp.pharm.mssm.edu/Enricher), a web-based platform for gene set enrichment, was used for performing the gene ontology (GO) and Kyoto Encyclopedia of Genes and Genomes (KEGG) analyses. The GO analysis included three ontologies: biological process (BP), cellular component (CC), and molecular functions (MF) [19]. Next, the data from the KEGG analysis was used for identifying the pathways involved in COVID-19 infection that were common with MM and DLBCL. The *P* value of < 0.05 and a count of ≥2 were used as significance and enrichment thresholds, respectively, in the GO and KEGG analyses.

### 2.4. The Protein-Protein Interaction Network Analysis

The PPI network of the common DEGs was constructed using the Search Tool for the Retrieval of Interacting Genes (String) database (https://string-db.org/; version 11.5) [20]. The PPI network was constructed using the relationship pairs with a combined score of >0.4. Several topological characteristics, including degree centrality (DC), eigenvector centrality (EC), and closeness centrality (CC), of the nodes (proteins) in the PPI network, were calculated using the CytoNCA plugin provided in the Cytoscape software [21](http://apps.cytoscape.org/apps/cytonca) to screen and reveal the hub genes.

### 2.5. Identification of the Hub Genes

The constructed PPI network included nodes, edges, and their connections, and the most overlapped genes were regarded as hub genes. CytoHubba (http://apps.cytoscape.org/apps/cytohubba), a plugin provided in the Cytoscape software, was employed to select the significant modules and the top-ranking genes. The key clusters were obtained using the Molecular Complex Detection (MCODE) application [22]. CytoHubba comprises 12 featured algorithms, among which, one of the finest is Maximal Clique Centrality (MCC) [23]. The MCC algorithm was used in the present study to identify the top five genes (hub genes) from the PPI network.

### 2.6. Constructing the miRNAs-Genes and Transcription Factors-Gene Networks

The miRTarBase [24], StarBase [25], and TargetScan [26] databases were used for the prediction of the target miRNAs of the common DEGs. In order to achieve better prediction accuracy, the miRNAs predicted based on all three databases were used in the prediction analysis. The Enrichr database (https://maayanlab.cloud/Enrichr/) was then used for predicting the TFs (transcription factors) that targeted the common DEGs. *P* value ≤ 0.05 was selected as the threshold. Finally, Cytoscape was employed to visualize the constructed miRNA-TF-mRNA regulatory network.

### 2.7. Identification of Potential Drug Candidates

The prediction of the protein-drug interaction (PDI) and the identification of drug molecules was crucial for the present study. The drug molecule was identified based on the common DEGs of COVID-19, MM, and DLBCL using the Drug Signatures database (DSigDB) in Enrichr. The drug target related to these DEGs was identified using an online tool DSigDB [27]. This database contained 22,527 gene sets and served as an efficient tool for accessing the DSigDB database via Enrichr under Diseases/Drugs function.

### 2.8. Analysis of the Gene-Disease Associations

The DisGeNET database is concerned with gene-disease associations, synchronizing connections of various origins featuring diverse biomedical aspects of the disease. The database provides novel insights into human genetic diseases [28]. In the present study, the gene-disease relationship was assessed using DSigDB through NetworkAnalyst to reveal the disorders associated with the identified common DEGs and their chronic complications.

## 3. Results

### 3.1. Identification of the DEGs and Common DEGs among MM, DLBCL, and COVID-19

**Figure 1:**
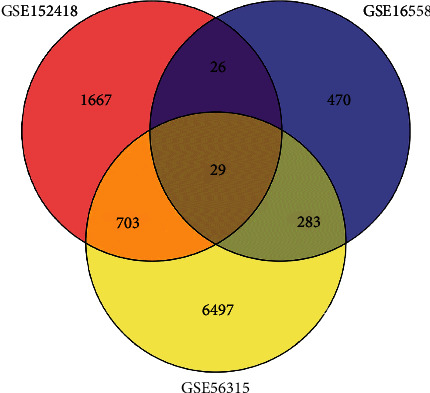
Two microarray datasets and one RNA-seq dataset containing the data of MM (GSE16558), DLBCL (GSE56315), and SARS-CoV-2 (GSE152418) patients were used for revealing 29 common DEGs among SARS-CoV-2, CLL, and DLBCL.

In order to understand the relationships and consequences of MM and DLBCL with COVID-19, NCBI datasets comprising human RNA-seq and microarray data were used for classifying the disordered genes with sequences related to COVID-19, MM, and DLBCL. These RNA-seq and microarray datasets were subjected to the DESeq2 tool in the R package and also to the Limma package with Benjamin-Hochberg correction to reduce the false discovery rate. Initially, 16558 differential genes were identified for COVID-19, which comprised 169 upregulated and 639 downregulated genes. These DEGs were then subjected to statistical analysis of significant differences to reveal the most significant DEGs common with MM and DLBCL. This revealed 56315 DEGs (3767 upregulated and 3745 downregulated) with the MM dataset and 152428 DEGs (2250 upregulated and 175 downregulated) with the DLBCL dataset. Jvenn was employed for the Venn analysis, followed by the cross-comparison analysis, which together revealed the common DEGs among MM, DLBCL, and SARS-CoV-2 databases. The three diseases are reported to be interrelated based on one or more common genes in previous studies as well [29].  Figure 1 presents the retrieved common DEGs among the three datasets along with the cumulative comparative evaluation.

### 3.2. Gene Ontology and Pathway Enrichment Analysis

**Figure 2:**
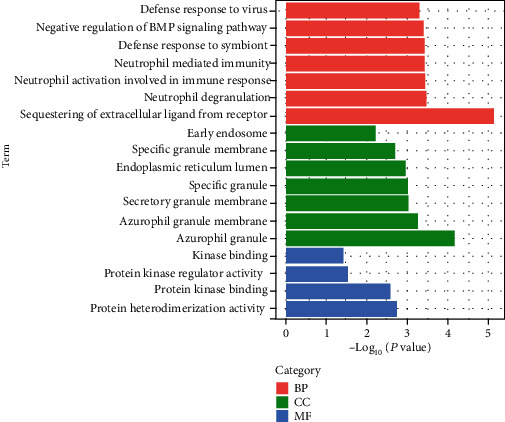
Gene ontology analysis of the common DEGs among SARS-CoV-2, MM, and DLBCL, performed using Enricher. The pink bars indicate the biological processes (BP), the green bars represent the molecular functions (MF), and the blue bars indicate the cellular components (CC).

The gene ontology and pathway enrichment analyses were performed using Enrichr to evaluate the biological significance and the enriched pathways of the common DEGs identified in the present study. Gene ontology considers the functions and components of genes and also provides massive measurable knowledge resources. An ontology defines an information body theoretically in a given context. Ontology and annotation are used for implementing a complete biological structure model, which assists in biological applications [30]. Gene ontology analysis is divided into three sections: biological process, cellular component, and molecular function. The GO database was selected as the annotation source in the present study.  Figure 2 illustrates the complete ontological analysis linearly along with its three categories in the form of a bar graph. The top 5 GO biological processes revealed for the DEGs identified in the present study were as follows: sequestering of the extracellular ligand iron receptor, neutrophil degradation, neutrophil activation and immune response, neutrophil-mediated immunity, and defense response to the symbiont. These processes suggested that these DEGs might have key roles in the regulation of neutrophil activity and, therefore, in neutrophil-associated immunity.

**Figure 3:**
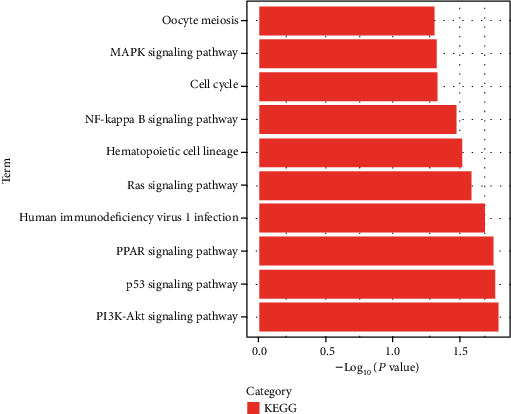
Kyoto Encyclopedia of Genes and Genomes (KEGG) pathway analysis of the common DEGs among COVID-19, MM, and DLBCL.

Pathway analysis reveals the connections with different diseases at the fundamental molecular or biological level [31]. The pathway enrichment analysis conducted in the present study for the identified DEGs common among COVID-19, MM, and DLBCL revealed the pathways of interest from the KEGG database.  Figure 3 depicts the bar graph representation of the results of the pathway enrichment analysis. According to the KEGG analysis, the DEGs were enriched the most in the PI3K-Akt pathway, p53 signaling pathway, PPAR signaling pathway, and RAS pathway. This highlighted the close association of these DEGs with senescence and apoptosis.

### 3.3. Classification of Hub Proteins and the Submodule

**Figure 4:**
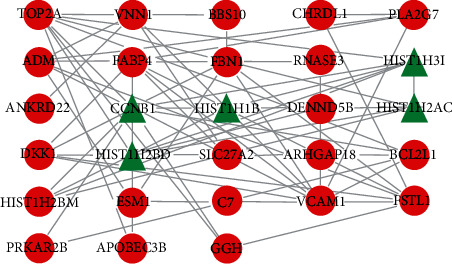
The hub genes identified from the PPI network using the CytoHubba plugin in Cytoscape. The hub genes were obtained through the latest MCC procedure of the CytoHubba plugin. The green triangle presents the top 5 hub genes and their interactions with other molecules.

STRING was employed to examine the PPI network visualized in Cytoscape in terms of anticipating the connections and linked pathways of common DEGs. The majority of the interconnected nodes in the PPI network were identified as hub genes. The clusters were obtained using the MCODE application. CytoHubba was employed to screen the significant genes based on topological algorithms [23]. The PPI enrichment *P* value was <1.0 × 10^−16^, which indicated that these proteins exhibited at least partial bioconjugation as a group, with interactions between these proteins stronger than those formed with other random proteins. In total, 28 nodes were identified in the PPI network analysis using the CytoHubba plugin in Cytoscape. Among these 28 nodes, the top 5 (17.24%) DEGs were selected as the most significant genes: CCNB1, HIST1H1B, HIST1H3I, HIST1H2AC, and HIST1H2BD. These hub genes could be utilized as biomarkers for disease prediction and may reveal novel therapeutic targets for disease treatment.  Figure 4 presents a submodule network constructed using the CytoHubba plugin. The expansion network with the interacting hub genes identified from the PPI network is depicted in Figure 4.

### 3.4. Determination of the Regulatory Signatures

**Figure 5:**
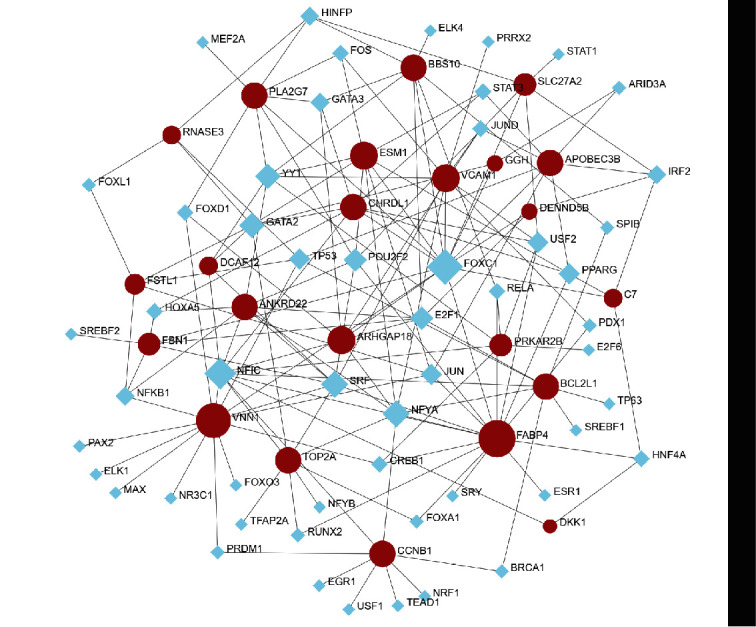
The DEG-TF regulatory interaction network obtained using NetworkAnalyst. The blue squares indicate the TFs, while the red circles represent the interactions of the gene symbols with the TFs. Network Analyst was also employed to retrieve the coherent regulatory interaction network of DEG-TFs. In this case, the TFs are indicated by blue square nodes, while the gene symbols are depicted using red circular nodes.

**Figure 6:**
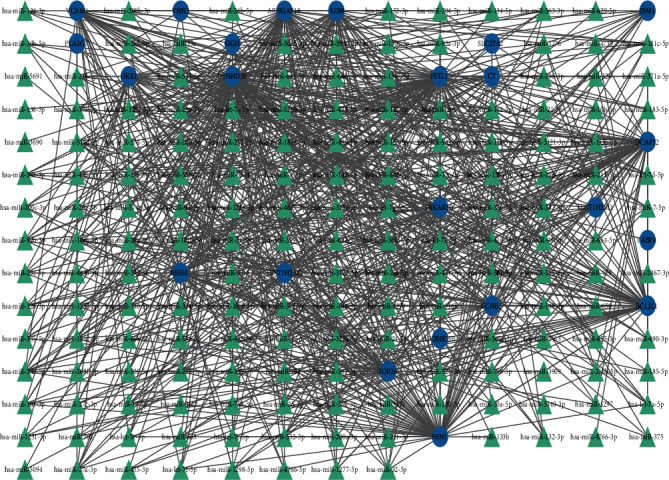
The interconnected regulatory interaction network of DEGs-miRNAs. The green triangles indicate the miRNAs, and the gene symbols that interact with these miRNAs are indicated by blue circles.

In order to verify the substantial changes that occur at the transcriptional level and better understand the regulatory molecules or common DEGs related to the determined hub genes, a network-based approach was adopted to predict the relevant transcriptional factors (TFs) and posttranscriptional regulatory factors (miRNAs). The interactions among the TFs, miRNA regulators, and common DEGs are illustrated in Figure 5. Next, the interactions of the miRNA regulators with the common DEGs are presented in Figure 6. Network analysis was performed to assess the interaction between the TFs and miRNAs of the genes, which revealed 54 TFs and 23 miRNA regulatory signatures involved in the regulation of more than one common DEG, indicating a strong interaction among them.

### 3.5. Identification of the Candidate Drugs

Evaluation of the interaction between proteins and drugs is crucial for understanding the structural basis of receptor sensitivity [29, 32]. In the present study, 10 potential drug molecules were selected from the DSigDB database in Enrichr based on the transcriptome signatures as the potential drugs to target the identified common DEGs among MM, DLBCL, and COVID-19. The top 10 chemical compounds were selected based on their *P* value and could serve as drugs for targeting the common DEGs among the three diseases.  Table 1 lists the common DEGs along with their corresponding potential effective drugs obtained from the DSigDB database.

### 3.6. Identification of Disease Association

**Figure 7:**
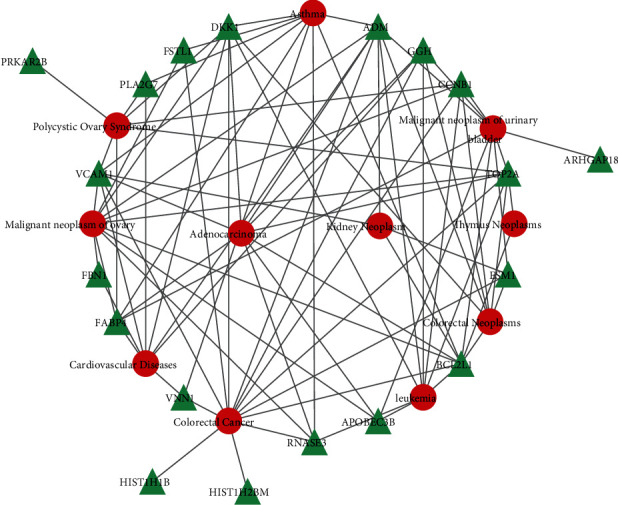
The gene-disease association network illustrates the disorders related to the common DEGs. The diseases are indicated by red circles, and the corresponding genetic symbols are indicated by green triangles.

Several disorders may be connected via one or more common genes [29]. Identification of these connections between genes and diseases would facilitate designing therapeutic strategies against these diseases [33]. NetworkAnalyst was employed in the present study to analyze the gene-disease associations. Asthma, polycystic ovary, malignant neoplasm of the ovary, cardiovascular diseases, adenocarcinoma, colorectal cancer, kidney neoplasm, malignant neoplasm of the urinary bladder, thymus neoplasms, colorectal neoplasms, and leukemia were revealed to be related to the hub genes among MM, DLBCL, and COVID-19 identified in the present study. Among these, the top 10 diseases were selected based on the *P* value. The relationships between genes and diseases are depicted in Figure 7.

## 4. Discussion

Multiple myeloma and diffuse large B-cell lymphoma are hematological malignancies [7]. Patients with these two diseases are more likely to be infected with COVID-19 and reportedly suffer from a further severe disease because of their reduced immune function, thereby presenting a significantly higher mortality rate [34]. The present study aimed at investigating the genes expressed among MM, DLBCL, and COVID-19 patients in the peripheral blood and accordingly select representative molecular targets that could be utilized as potential biomarkers of the risk of COVID-19 disease. In biomedical research, expression profiling using an array of datasets is considered a valuable resource for identifying biomarker candidates for various diseases [35]. In order to demonstrate that the 29 common DEGs identified in the present study have comparable expression patterns in MM, DLBCL, and SARS-CoV-2, these DEGs were analyzed using transcriptomics. The Gene Ontology (GO) analysis was used for evaluating and screening the 29 common DEGs based on their functions and *P* values to further understand their biological significance in the pathogenesis of MM, DLBCL, and COVID-19.

GO analysis reflects gene regulation based on a generic theoretical model that assists in identifying genes and their interrelationships, thereby contributing to the biological knowledge regarding gene activities and their regulation in terms of different ontological categories [36]. The online tool Enrichr used in the present study includes three ontologies in the GO analysis—biological process (BF), cellular component (CC), and molecular function (MF). The analysis was performed using the GO database as the annotation source for the ontological processes [37]. In the biological process, negative regulation of the BMP signaling pathway was revealed as a significant GO term. The BMP signaling pathway stimulates the differentiation of mesenchymal stem cells (MSCs) into osteoblasts in MM patients through the upregulation of EMX2. One of the reasons for bone deterioration in MM is the severe impairment of osteoblast activity, which provides a reasonable basis for clinical therapy against MM [38]. In DLBCL patients, the BMP signaling pathway activates SMAD5, which plays a tumor-suppressive role in lymphoid neoplasms [39]. Interestingly, SARS-CoV-2 is also related to the BMP signaling pathway [40]. Therefore, it was speculated that this pathway could be used as a promising cotherapeutic target in the treatment of MM or DLBCL patients with COVID-19 infection.

Pathway analysis is considered the best approach to reflect the internal changes occurring during a biological process. Therefore, the KEGG pathway of the 29 common DEGs among MM, DLBCL, and COVID-19 was conducted in the present study. Six important signaling pathways were revealed in the analysis: MAPK, NF-*κ*B, Ras, PPAR, P53, and PI3K-Akt pathways. The MAPK signaling pathway is crucial for the proliferation of B lymphocytes. The four major branches of the MAPK pathway are ERK(Ras/Raf/MEK/ERK), JNK, P38/MAPK, and ERK5. While JNK and p38 share similar roles and are related to inflammation, apoptosis, and proliferation, the ERK branch is primarily related to the growth and differentiation of duct cells and is reportedly activated by the well-recognized Ras/Raf protein [41]. SB203580, an inhibitor of p38, and U0126, an inhibitor of ERK, were reported to substantially inhibit cell proliferation and tumor growth in patients with DLBCL [42]. The use of 6-amino-4-quinazoline inhibitors was reported to successfully suppress the IL-6-induced MAPK pathway [43] and is, therefore, considered an important method in MM therapy. In SARS-COV-2 infection, inflammation is induced via the p38 MAPK pathway. Therefore, blocking this pathway could relieve COVID-19 infection, although additional preclinical studies are warranted to verify this speculation [44]. Therefore, the MAPK pathway is considered an important link between patients with MM or DLBCL and COVID-19 patients and could serve as a novel therapeutic route in the treatment of these patients.

Furthermore, the NF-*κ*B pathway is reported to induce an antiapoptotic and proproliferative gene program [45]. The NF-*κ*B pathway could, therefore, be inhibited to increase the survival rates in patients with MM and DLBCL, while also serving as a target for alleviating disease severity in COVID-19 patients [46]. The p53 pathway is involved in apoptosis, and the downregulation of this pathway might promote the replication of coronavirus [47]. The activator of p53 could, therefore, be used in the therapy for COVID-19 in the future. The components of the p53 pathway include MYC, RAS, ARF, MDM2, ATM, and TP53. TP53 deletions and mutations are common in DLBCL patients, while MYC additions are common in MM patients. Furthermore, these two are inversely correlated with survival in MM and DLBCL patients [48]. Therefore, studying the inhibitors of the p53 pathway could reveal certain novel strategies for the care of patients with MM or DLBCL who are infected with COVID-19 in the future. The patients of COVID-19, MM, and DLBCL may also be treated with specific inhibitors of the PI3K/Akt/mTOR pathway [49, 50]. In summary, the MAPK, NF-*κ*B, P53, and PI3K-Akt pathways were revealed to be associated with the patients of MM or DLBCL with COVID-19 infection and could, therefore, be studied in future research to reveal novel targets for COVID-19 therapy.

The PPI network constructed in the present study using the identified DEGs revealed 5 hub proteins associated with MM, DLBCL, and COVID-19: CCNB1, HIST1H2BD, HIST1H1B, HIST1H2AC, and HIST1H3I. The protein CCNB1 is involved in the cell cycle and mitosis and may be utilized as a predictor of high-risk disorders and poor prognosis of gene expression in MM patients [51, 52]. The downregulation of cell cycle-related protein CCNB1 using the drug Glaucocalyxin A (GLA) could inhibit cell proliferation while increasing the expression of p21 in MM cell lines. Therefore, the use of this protein could serve as a potential treatment method for MM patients [53]. Meanwhile, based on the currently available data on COVID-19 patients, CCNB1 may also be used as a potential biomarker of COVID-19 in peripheral blood mononuclear cells (PBMCs), thereby contributing to the development of drugs for the treatment of COVID-19 disease [54]. The protein HIST1H2BD is expressed in myeloma cells. The use of the drug ixazomib reportedly inhibited myeloma cell growth by significantly increasing HIST1H2BD expression and reducing UBE2K expression [55]. The roles of most of the core gene-encoded proteins in DLBCL patients have not been elucidated experimentally so far [56], and could, therefore, be a focus of future investigations in this regard. In conclusion, the hub genes identified in the present study could be used as prognostic biomarkers and therapeutic targets in the treatment of COVID-19 disease.

Furthermore, the TF-genes and miRNAs interactions were also explored in the present study to reveal the transcriptional and posttranscriptional regulators of the common DEGs for the three diseases studied. Knowledge of TFs and miRNAs is important for understanding the development of a disorder. The present study revealed several such connections among the mutual DEGs, TFs, and miRNAs. For instance, the identified TFs, namely, MEF2A, FOS, STAT1, STAT3, JUND, YY1, IRF2, ARID3A, SPIB, TP53, RELA, E2F1, NFKB1, NR3C1, FOXO3, PRDM1, NRF1, and ESR1 are related to various hematological diseases. In addition, several of the miRNAs are related to MM and DLBCL patients, such as the involvement of miR-124-3p dysregulation in different kinds of cancers. Targeting the LINC01234/miR-124-3p/GRB2 axis reportedly increased the expression of miR-124-3p and inhibited the expression of the GRB2 protein through the downregulation of LINC01234, which ultimately decreased the proliferation of the MM tumor cells [57]. Moreover, miR-215-3p acts as a tumor suppressor in MM, inhibiting cell proliferation and promoting apoptosis in MM cells by targeting RUNX1 and deactivating the PI3K/AKT/mTOR pathway [58]. These findings could provide a novel direction to the research on developing targeted therapies for patients with MM in the future. Another miRNA named miR-665 could be used as an antitumor agent in DLBCL. The overexpression of miR-665 inhibits cell proliferation and invasion while promoting DLBCL apoptosis via two target sites, MYC and LASP1. MYC is, therefore, a target gene of miR-665 and could be utilized as an indicator of the prognosis of DLBCL. In addition, miR-665 may prevent the progression of DLBCL through the downregulation of LASP1 and MYC [59]. The molecular mechanism underlying the impact of miR-665 on DLBCL could, therefore, be another focus of future research. The present study revealed certain genes that were significantly associated with MM and DLBCL, such as those encoding miR-152-3p, miR-128-3p, miR-335-5p, miR-340-5p, miR-101-3p, smiR-17-5p, miR-455-3p, miR-9-5p, miR-155-5p, miR-21-5p, and miR-375. The majority of these miRNAs are related to cancer tissues and cause various types of cancers in humans, particularly hematological malignancies.

A gene-disease (GD) relationship analysis was also conducted in the present study to predict the associations of the significant DEGs with various disorders. The experimental results revealed a variety of disorders that developed in COVID-19, including those of the lung, kidney, colorectal, thymus, ovary, cardiac, and various types of leukemias. Two examples of respiratory diseases are pneumonia and acute respiratory distress, which are the primary complications of COVID-19 [60]. The relationship between the severity of COVID-19 disease and allergic diseases, including asthma, remains to be elucidated so far [61]. When the COVID-19 virus infects the lungs, it could reach the kidney via blood circulation, causing kidney tubular injury as another primary manifestation of SARS-CoV-2 infection. The major diagnostic symptom of this renal involvement is proteinuria [62]. The most common cardiac abnormality observed so far in COVID-19 infection is acute heart injury, which accounts for approximately 8% of all cases [63]. The primary transmission route for the COVID-19 virus is respiratory droplet transmission, while the fecal-oral transmission route is also observed in certain cases. Patients presenting initially with gastrointestinal symptoms are, therefore, considered potential spreaders of infection. In addition to the vulnerability of the digestive tract to COVID-19 infection, up to 60% of the infected people also develop liver disease [64].

Formerly, a variety of chemicals and drugs were used in the treatment of COVID-19 disease. For instance, Baicalein was used for enhancing respiratory function, preventing pulmonary inflammatory cell infiltration, and reducing the levels of IL-1*β* and TNF-*α* in the serum. Baicalein is considered a promising medicine for COVID-19 [65]. In addition, Trichostatin A could limit SARS-COV-2 replication and enhance the antiviral activity when used in combination with other drugs [66]. Green tea polyphenols have also exhibited beneficial effects in several pathological disorders, such as cancer, diabetes, and cardiovascular disorders. These are used for alleviating blood pressure, blood fat, cholesterol levels, and blood sugar levels to prevent atherosclerosis and protect from cardiovascular disorders [67]. Desferrioxamine reportedly increases the ferritin levels in the acute phase of SARS-COV-2 infection and decreases liver injury complications [68]. Another drug named LY-294002 inhibits the PI3K/AKT signaling pathway and is used for treating various forms of cancer, particularly liver cancer, gastric cancer, and leukemia [69–71]. Moreover, both imatinib and dasatinib are inhibitors of the tyrosine kinase signaling pathways, specifically the ABL1 pathway. The ABL1 pathway is related to cell differentiation, cell adhesion, and cell stress. Overactivation of the ABL1 pathway leads to cancer development. Imatinib and dasatinib are used for the treatment of leukemias and the effective suppression of MERS-CoV and SARS-CoV [72, 73]. CoCl_2_ is used for the therapy of anemia in patients with renal failure [74]. Another drug named fenofibrate is used for reducing dyslipidemia and downgrading COVID-19 infections via two molecular routes—blockage of the virus metabolism and replication and inhibition of the ACE2 receptor-binding domain [75]. Besides hematological malignancies, a wide variety of diseases, such as kidney diseases, respiratory diseases, cardiac disorders [76, 77], brain conditions, and different types of cancers, are related to COVID-19 infection [78]. Consequently, the above-stated medications are effective in treating COVID-19 infections as well.

## 5. Conclusions

The present study revealed the relationships among MM, DLBCL, and COVID-19 genes based on a transcriptional analysis. The three datasets corresponding to the three diseases were used for identifying the DEGs, among which the common DEGs for the three diseases were retrieved to understand the disease responses based on immune cells in MM, DLBCL, and COVID-19. The bioinformatics approach revealed that patients with MM and DLBCL have a high risk of SARS-CoV-2 infection. A total of 29 common interrelated genes were retrieved from these datasets in the present study. Next, a PPI network was constructed to identify the common genes and select the 5 most critical hub genes. Several drug molecules and drug-target linkages with these hub genes were revealed through a search of the DSigDB database. This approach of analyzing the association of COVID-19 with MM and DLBCL could be used for predicting COVID-19 infection and disease severity in patients with MM and DLBCL, thereby facilitating a reduction in the risk of SARS-CoV-2 infection in these patients. So far, the literature on the risk of and the disease factors associated with the novel COVID-19 disease is scarce. The present study, therefore, provides the above-described approach to analyzing COVID-19. Currently, a few vaccines are available for the prevention of COVID-19 disease. However, these vaccines may not be entirely effective, particularly against different variants of SARS-CoV-2 that keep emerging. Therefore, further effective COVID-19 vaccines are required, which could be facilitated by transcriptome analysis. A revelation of the common pathways and molecular indicators among MM, DLBCL, and COVID-19 would assist in understanding the relationship between SARS-CoV-2, MM, and DLBCL. In this context, five relevant hub genes for these diseases were identified in the present study. In addition, the TFs and miRNAs identified in the present study were associated with various forms of hematological disorders. Therefore, based on the genes identified in the present study, a novel therapeutic target could be identified to develop an improved COVID-19 vaccine. In summary, the present study highlighted the potential therapeutic targets for developing future treatments for the three diseases. In addition, the identified hub genes could be useful in developing novel, efficient vaccines against COVID-19 disease.

## Figures and Tables

**Table 1:** Recommended drugs that are listed for COVID-19.

Name	*P* value	Chemical formula
Genistein CTD 00007324	5 46*E* − 08	C_15_H_10_O_5_
Trichostatin A CTD 00000660	1 126*E* − 06	C_17_H_22_N_2_O_5_
Epigallocatechin gallate CTD 00002033	2 63E 06	C_2_H_18_O_11_
Deferoxamine MCF7 DOWN	3 07*E* − 06	C_25_H_48_N_6_O_8_
LY-294002 HL60 UP	4 14*E* − 06	C_19_H_17_NO_3_
Dasatinib CTD 00004330	5 82*E* − 06	C_22_H_26_CIN_7_O_2_S
Isoprenaline HL60 UP	5 87*E* − 06	C_11_H_17_NO_3_
7646 79 9 CTD 00000928	5 96*E* 06	CI_2_CO
Resveratrol CTD 00002483	7 47*E* − 06	C_14_H_12_O_3_
Fenofibrate CTD 00006620	8 35*E* − 06	C_20_H_21_CIO_4_

## Data Availability

Publicly available datasets were analyzed in this study. These data can be found here in National Center for Biotechnology Information (NCBI), Gene Expression Omnibus (GEO), https://www.ncbi.nlm.nih.gov/geo/, GSE152418, GSE16558, and GSE56315.
