# Supplementary material for: Identifying the Effect of COVID-19 Infection in Multiple Myeloma and Diffuse Large B-Cell Lymphoma Patients Using Bioinformatics and System Biology
Source: Comput Math Methods Med. 2022 Nov 23;2022:7017317. doi: 10.1155/2022/7017317 (PMC9711963; doi:10.1155/2022/7017317)
Supplement: Supplementary Materials — The Supplementary Material for this article can be found in a word named supplementary data. [file 7017317.f1.docx]

Table S1. Details of GO CC for SARS-CoV-2 ,MM and DLBCL.

| Term | P-values | Genes |
| --- | --- | --- |
| azurophil granule (GO:0042582) | 6.670085000849696E-5 | VNN1;CEACAM6;GGH;RNASE3;CEACAM8 |
| azurophil granule membrane (GO:0035577) | 5.455315009318622E-4 | VNN1;CEACAM6;CEACAM8 |
| secretory granule membrane (GO:0030667) | 9.252288728793671E-4 | MS4A3;VNN1;CEACAM6;CEACAM8;SLC27A2 |
| specific granule (GO:0042581) | 9.795249274081729E-4 | MS4A3;GGH;CEACAM8;SLC27A2 |
| endoplasmic reticulum lumen (GO:0005788) | 0.0011026398791295158 | TF;CHRDL1;SLC27A2;FSTL1;FBN1 |
| specific granule membrane (GO:0035579) | 0.002014713593609096 | MS4A3;CEACAM8;SLC27A2 |
| early endosome (GO:0005769) | 0.006131591730120902 | TF;PARM1;VCAM1;DKK1 |
| intracellular organelle lumen (GO:0070013) | 0.008291035057751405 | CCNB1;TF;GGH;CHRDL1;SLC27A2;FSTL1;FBN1 |
| filopodium (GO:0030175) | 0.011122382711878437 | KITLG;VCAM1 |
| meiotic spindle (GO:0072687) | 0.013675819832555556 | FBXO5 |
| membrane attack complex (GO:0005579) | 0.016388876219180396 | C7 |
| low-density lipoprotein particle (GO:0034362) | 0.019094606376572693 | PLA2G7 |
| extrinsic component of external side of plasma membrane (GO:0031232) | 0.02179302948367254 | TF |
| HFE-transferrin receptor complex (GO:1990712) | 0.02179302948367254 | TF |
| actin-based cell projection (GO:0098858) | 0.0219196196602785 | KITLG;VCAM1 |
| azurophil granule lumen (GO:0035578) | 0.025484900745099414 | GGH;RNASE3 |
| microfibril (GO:0001527) | 0.029844648447595826 | FBN1 |
| integral component of peroxisomal membrane (GO:0005779) | 0.035176210749711605 | SLC27A2 |
| podosome (GO:0002102) | 0.035176210749711605 | VCAM1 |
| intrinsic component of peroxisomal membrane (GO:0031231) | 0.037831194118660955 | SLC27A2 |
| supramolecular fiber (GO:0099512) | 0.05099889303736216 | FBN1 |
| high-density lipoprotein particle (GO:0034364) | 0.05099889303736216 | PLA2G7 |
| secretory granule lumen (GO:0034774) | 0.05616943517347117 | TF;GGH;RNASE3 |
| endosome membrane (GO:0010008) | 0.06009289039106718 | TF;FLT3;DKK1 |
| lysosomal membrane (GO:0005765) | 0.06232740231766286 | VNN1;CEACAM6;CEACAM8 |
| sodium channel complex (GO:0034706) | 0.06656673915811412 | SCNN1B |
| vacuolar lumen (GO:0005775) | 0.07250206578980918 | GGH;RNASE3 |
| tertiary granule (GO:0070820) | 0.07485194777603997 | GGH;CEACAM8 |
| cyclin-dependent protein kinase holoenzyme complex (GO:0000307) | 0.07934818460888594 | CCNB1 |
| Cul4-RING E3 ubiquitin ligase complex (GO:0080008) | 0.08944948211911666 | DCAF12 |
| late endosome (GO:0005770) | 0.2551045656503671 | TF;PARM1 |
| serine/threonine protein kinase complex (GO:1902554) | 0.09695396842829543 | CCNB1 |
| spindle (GO:0005819) | 0.09788896123793156 | FBXO5;CDC6 |
| microbody membrane (GO:0031903) | 0.13357498408436996 | SLC27A2 |
| basement membrane (GO:0005604) | 0.13357498408436996 | FBN1 |
| peroxisomal membrane (GO:0005778) | 0.1359638603430485 | SLC27A2 |
| tertiary granule lumen (GO:1904724) | 0.14072222881779534 | GGH |
| microvillus (GO:0005902) | 0.1454548662281174 | VCAM1 |
| specific granule lumen (GO:0035580) | 0.15717478081657915 | GGH |
| clathrin-coated endocytic vesicle membrane (GO:0030669) | 0.17331793667880604 | TF |
| cation channel complex (GO:0034703) | 0.18240588726064513 | SCNN1B |
| tertiary granule membrane (GO:0070821) | 0.18240588726064513 | CEACAM8 |
| voltage-gated potassium channel complex (GO:0008076) | 0.18240588726064513 | KCNA2 |
| lipid droplet (GO:0005811) | 0.19139572828895293 | FABP4 |
| P-body (GO:0000932) | 0.19807435058693812 | APOBEC3B |
| potassium channel complex (GO:0034705) | 0.19807435058693812 | KCNA2 |
| nuclear chromosome (GO:0000228) | 0.20469880592331144 | TOP2A |
| clathrin-coated endocytic vesicle (GO:0045334) | 0.20908522987063624 | TF |
| intracellular non-membrane-bounded organelle (GO:0043232) | 0.21197754483642553 | TOP2A;VCAM1;FABP4;FBXO5;CDC6 |
| clathrin-coated vesicle membrane (GO:0030665) | 0.2199476354622725 | TF |
| early endosome membrane (GO:0031901) | 0.23490930472582544 | DKK1 |
| cytoplasmic vesicle lumen (GO:0060205) | 0.2721004612419189 | TF |
| cytoplasmic vesicle membrane (GO:0030659) | 0.28095122419302415 | TF;FLT3 |
| mitotic spindle (GO:0072686) | 0.28211241656016706 | CDC6 |
| intrinsic component of endoplasmic reticulum membrane (GO:0031227) | 0.2840985210272574 | SLC27A2 |
| mitochondrial outer membrane (GO:0005741) | 0.29394840371672587 | BCL2L1 |
| mitochondrial envelope (GO:0005740) | 0.2959023524817266 | IFI6 |
| peroxisome (GO:0005777) | 0.2978509918817559 | SLC27A2 |
| integral component of endoplasmic reticulum membrane (GO:0030176) | 0.3245823217168106 | SLC27A2 |
| organelle outer membrane (GO:0031968) | 0.3245823217168106 | BCL2L1 |
| recycling endosome (GO:0055037) | 0.3301791059497975 | TF |
| cullin-RING ubiquitin ligase complex (GO:0031461) | 0.35211445682454806 | DCAF12 |
| endocytic vesicle membrane (GO:0030666) | 0.35391023593741583 | TF |
| chromosome (GO:0005694) | 0.3574871455507493 | TOP2A |
| integral component of plasma membrane (GO:0005887) | 0.37074398866424424 | VCAM1;C7;FLT3;SCNN1B;KCNA2 |
| mitochondrial membrane (GO:0031966) | 0.3708864482135022 | IFI6;BCL2L1 |
| endocytic vesicle (GO:0030139) | 0.4072188691837333 | TF |
| axon (GO:0030424) | 0.4314387106959538 | KCNA2 |
| integral component of organelle membrane (GO:0031301) | 0.44704270242141475 | SLC27A2 |
| vesicle (GO:0031982) | 0.4652131906567196 | TF |
| dendrite (GO:0030425) | 0.526959319830245 | KCNA2 |
| actin cytoskeleton (GO:0015629) | 0.5840251969285896 | VCAM1 |
| mitochondrial inner membrane (GO:0005743) | 0.5977641164749024 | IFI6 |
| microtubule cytoskeleton (GO:0015630) | 0.6011286287398175 | FBXO5 |
| nucleolus (GO:0005730) | 0.6037002238033192 | TOP2A;CDC6 |
| nuclear lumen (GO:0031981) | 0.6126578709439398 | TOP2A;CDC6 |
| organelle inner membrane (GO:0019866) | 0.6175410920789544 | IFI6 |
| mitochondrial matrix (GO:0005759) | 0.6196786997717256 | CCNB1 |
| bounding membrane of organelle (GO:0098588) | 0.6286891493666579 | TF;FLT3 |
| collagen-containing extracellular matrix (GO:0062023) | 0.6523271473223632 | FBN1 |
| focal adhesion (GO:0005925) | 0.6590933672244434 | ITGA8 |
| cell-substrate junction (GO:0030055) | 0.665730254332735 | ITGA8 |
| neuron projection (GO:0043005) | 0.7883406994929107 | KCNA2 |
| nucleus (GO:0005634) | 0.8191618535127099 | TOP2A;DUSP4;SPIC;CCNB1;FABP4;GGH;FBXO5;CDC6;ZNF14;APOBEC3B |
| endoplasmic reticulum membrane (GO:0005789) | 0.8641819040485825 | SLC27A2 |
| intracellular membrane-bounded organelle (GO:0043231) | 0.8799318566328262 | TOP2A;DUSP4;SPIC;CCNB1;PARM1;FABP4;GGH;FBXO5;CDC6;ZNF14;APOBEC3B |

Table S2. Details of GO BP for SARS-CoV-2 ,MM and DLBCL.

| Term | P-values | Genes |
| --- | --- | --- |
| sequestering of extracellular ligand from receptor (GO:0035581) | 7.010638301606187E-6 | CHRDL1;FSTL1;FBN1 |
| regulation of mitotic metaphase/anaphase transition (GO:0030071) | 4.8919217375186E-5 | CCNB1;FBXO5;CDC6 |
| positive regulation of chromosome segregation (GO:0051984) | 2.057099035637798E-4 | CCNB1;CDC6 |
| sequestering of BMP from receptor via BMP binding (GO:0038098) | 2.057099035637798E-4 | CHRDL1;FSTL1 |
| heterophilic cell-cell adhesion via plasma membrane cell adhesion molecules (GO:0007157) | 2.0937561423390504E-4 | VCAM1;CEACAM6;CEACAM8 |
| neutrophil degranulation (GO:0043312) | 3.3326461019303374E-4 | MS4A3;VNN1;CEACAM6;GGH;RNASE3;CEACAM8;SLC27A2 |
| neutrophil activation involved in immune response (GO:0002283) | 3.503150808194479E-4 | MS4A3;VNN1;CEACAM6;GGH;RNASE3;CEACAM8;SLC27A2 |
| neutrophil mediated immunity (GO:0002446) | 3.635577793711669E-4 | MS4A3;VNN1;CEACAM6;GGH;RNASE3;CEACAM8;SLC27A2 |
| defense response to symbiont (GO:0140546) | 3.759559193133122E-4 | IFIT1B;IFI6;BCL2L1;APOBEC3B |
| negative regulation of BMP signaling pathway (GO:0030514) | 3.953067687192971E-4 | CHRDL1;FSTL1;FBN1 |
| defense response to virus (GO:0051607) | 4.901951038975145E-4 | IFIT1B;IFI6;BCL2L1;APOBEC3B |
| regulation of single stranded viral RNA replication via double stranded DNA intermediate (GO:0045091) | 0.0011044055879804073 | TOP2A;APOBEC3B |
| release of cytochrome c from mitochondria (GO:0001836) | 0.0011044055879804073 | IFI6;BCL2L1 |
| positive regulation of G2/M transition of mitotic cell cycle (GO:0010971) | 0.001366658495614331 | CCNB1;FBXO5 |
| positive regulation of cellular process (GO:0048522) | 0.0015537992021179567 | ESM1;KITLG;FLT3;CEACAM6;FBXO5;CDC6;DKK1 |
| regulation of mitotic cell cycle phase transition (GO:1901990) | 0.0017741594955385204 | CCNB1;PRKAR2B;FBXO5;CDC6 |
| positive regulation of cell cycle G2/M phase transition (GO:1902751) | 0.0018102215186427363 | CCNB1;FBXO5 |
| negative regulation of extrinsic apoptotic signaling pathway in absence of ligand (GO:2001240) | 0.0018102215186427363 | IFI6;BCL2L1 |
| negative regulation of signal transduction in absence of ligand (GO:1901099) | 0.0018102215186427363 | IFI6;BCL2L1 |
| regulation of extrinsic apoptotic signaling pathway in absence of ligand (GO:2001239) | 0.0026808978945138846 | IFI6;BCL2L1 |

Table S3. Details of GO MF for SARS-CoV-2 ,MM and DLBCL.

| Term | P-values | Genes |
| --- | --- | --- |
| protein heterodimerization activity (GO:0046982) | 0.0017741594955385204 | TOP2A;CEACAM6;CEACAM8;BCL2L1 |
| protein kinase binding (GO:0019901) | 0.002583629736233807 | TOP2A;DUSP4;CCNB1;PRKAR2B;FBXO5;BCL2L1 |
| BH domain binding (GO:0051400) | 0.016388876219180396 | BCL2L1 |
| BH3 domain binding (GO:0051434) | 0.016388876219180396 | BCL2L1 |
| 1-alkyl-2-acetylglycerophosphocholine esterase activity (GO:0003847) | 0.019094606376572693 | PLA2G7 |
| patched binding (GO:0005113) | 0.019094606376572693 | CCNB1 |
| vascular endothelial growth factor-activated receptor activity (GO:0005021) | 0.019094606376572693 | FLT3 |
| long-chain fatty acid binding (GO:0036041) | 0.019094606376572693 | FABP4 |
| cyclin-dependent protein serine/threonine kinase activator activity (GO:0061575) | 0.02179302948367254 | CCNB1 |
| ubiquitin ligase inhibitor activity (GO:1990948) | 0.02179302948367254 | FBXO5 |
| deoxycytidine deaminase activity (GO:0047844) | 0.02179302948367254 | APOBEC3B |
| calcium-independent phospholipase A2 activity (GO:0047499) | 0.02179302948367254 | PLA2G7 |
| cAMP-dependent protein kinase inhibitor activity (GO:0004862) | 0.02179302948367254 | PRKAR2B |
| oxidoreductase activity, acting on the CH-NH2 group of donors, oxygen as acceptor (GO:0016641) | 0.024484164739851953 | VCAM1 |
| ubiquitin-protein transferase inhibitor activity (GO:0055105) | 0.024484164739851953 | FBXO5 |
| MAP kinase tyrosine/serine/threonine phosphatase activity (GO:0017017) | 0.027168031335252564 | DUSP4 |
| protein tyrosine/threonine phosphatase activity (GO:0008330) | 0.027168031335252564 | DUSP4 |
| cAMP-dependent protein kinase regulator activity (GO:0008603) | 0.027168031335252564 | PRKAR2B |
| ligand-gated sodium channel activity (GO:0015280) | 0.027168031335252564 | SCNN1B |
| protein kinase regulator activity (GO:0019887) | 0.029827077157263297 | CCNB1;PRKAR2B |

Table S4. Details of KEGG for SARS-CoV-2 ,MM and DLBCL.

| Term | P-values | Genes |
| --- | --- | --- |
| PI3K-Akt signaling pathway | 0.016206729468657487 | KITLG;FLT3;ITGA8;BCL2L1 |
| p53 signaling pathway | 0.017225308933891778 | CCNB1;BCL2L1 |
| PPAR signaling pathway | 0.017672928580879252 | FABP4;SLC27A2 |
| Human immunodeficiency virus 1 infection | 0.020571297051512002 | CCNB1;BCL2L1;APOBEC3B |
| Ras signaling pathway | 0.025981436658664012 | KITLG;FLT3;BCL2L1 |
| Hematopoietic cell lineage | 0.03038926072393593 | KITLG;FLT3 |
| NF-kappa B signaling pathway | 0.03326301065542126 | VCAM1;BCL2L1 |
| Cell cycle 2/124 | 0.04574923442940984 | CCNB1;CDC6 |
| MAPK signaling pathway | 0.047124354803165805 | DUSP4;KITLG;FLT3 |
| Oocyte meiosis | 0.049102959369859304 | CCNB1;FBXO5 |
| Pantothenate and CoA biosynthesis | 0.056216290109268874 | VNN1 |
| Cell adhesion molecules | 0.06261457000936727 | VCAM1;ITGA8 |
| Folate biosynthesis | 0.06913688806581876 | GGH |
| Asthma | 0.08188377576047076 | RNASE3 |
| African trypanosomiasis | 0.09695396842829543 | VCAM1 |
| Aldosterone-regulated sodium reabsorption | 0.09695396842829543 | SCNN1B |
| Transcriptional misregulation in cancer | 0.09788896123793156 | FLT3;BCL2L1 |
| Ferroptosis | 0.10686555874711659 | TF |
| Lipid and atherosclerosis | 0.11810829981587277 | VCAM1;BCL2L1 |
| Ether lipid metabolism | 0.12636938033824105 | PLA2G7 |

Table S5. Details of recommended drugs for SARS-CoV-2 ,MM and DLBCL.

| Term | P-values | Genes |
| --- | --- | --- |
| genistein CTD 00007324 | 5.460547689590996E-8 | TOP2A;ESM1;CCNB1;VCAM1;GGH;DKK1;HIST1H1B;HIST1H2AC;HIST1H2BD;BCL2L1;FBN1;APOBEC3B |
| trichostatin A CTD 00000660 | 1.1212995185113808E-6 | VCAM1;DENND5B;ANKRD22;ARHGAP18;ADM;PLA2G7;FSTL1;DKK1;CCNB1;BBS10;PRKAR2B;SLC27A2;HIST1H2AC;HIST1H2BD;BCL2L1;FBN1;APOBEC3B |
| (-)-Epigallocatechin gallate CTD 00002033 | 2.62774868876846E-6 | TOP2A;HIST1H2BM;VCAM1;ARHGAP18;ADM;DKK1;CCNB1;BBS10;SLC27A2;HIST1H1B;HIST1H2AC;BCL2L1;FBN1 |
| deferoxamine MCF7 DOWN | 3.073014844063308E-6 | TOP2A;CCNB1;DKK1 |
| LY-294002 HL60 UP | 4.143191672683946E-6 | HIST1H3I;HIST1H2AC;HIST1H2BD |
| Dasatinib CTD 00004330 | 5.816713204108735E-6 | TOP2A;HIST1H2BM;ARHGAP18;DKK1;HIST1H1B;BCL2L1;APOBEC3B |
| (-)-isoprenaline HL60 UP | 5.8743888933297985E-6 | VNN1;HIST1H3I;PLA2G7;HIST1H2AC;HIST1H2BD |
| 7646-79-9 CTD 00000928 | 5.962635650195837E-6 | TOP2A;HIST1H2BM;ARHGAP18;ADM;FSTL1;DKK1;ESM1;CCNB1;FABP4;PRKAR2B;HIST1H3I;SLC27A2;HIST1H1B;HIST1H2AC;APOBEC3B |
| resveratrol CTD 00002483 | 7.467581827753399E-6 | TOP2A;CCNB1;VCAM1;FABP4;GGH;ADM;SLC27A2;HIST1H2AC;HIST1H2BD;BCL2L1;APOBEC3B |
| fenofibrate CTD 00006620 | 8.35454859587917E-6 | VCAM1;FABP4;PLA2G7;BCL2L1 |
| ZINC CTD 00007011 | 9.520402546470357E-6 | TOP2A;VCAM1;BBS10;VNN1;FABP4;C7;DENND5B;ADM;HIST1H2AC;BCL2L1;APOBEC3B |
| Capsaicin CTD 00005570 | 1.0722878881222885E-5 | CCNB1;VCAM1;FABP4;PLA2G7;BCL2L1 |
| COPPER CTD 00005706 | 1.2399406241868605E-5 | TOP2A;CCNB1;VCAM1;PRKAR2B;ADM;PLA2G7;SLC27A2;HIST1H1B;HIST1H2AC;HIST1H2BD;FBN1 |
| estradiol CTD 00005920 | 1.7121812863358625E-5 | TOP2A;HIST1H2BM;VCAM1;ANKRD22;ARHGAP18;GGH;ADM;PLA2G7;DKK1;CCNB1;VNN1;PRKAR2B;SLC27A2;HIST1H2AC;HIST1H2BD;BCL2L1;APOBEC3B |
| benzo[a]pyrene CTD 00005488 | 2.2656430571208842E-5 | TOP2A;HIST1H2BM;VCAM1;ARHGAP18;GGH;ADM;PLA2G7;DKK1;ESM1;CCNB1;VNN1;PRKAR2B;HIST1H1B;HIST1H2BD;BCL2L1;FBN1;APOBEC3B |
| rosiglitazone CTD 00003139 | 2.3102458524402678E-5 | CCNB1;VCAM1;FABP4;ADM;HIST1H2BD;BCL2L1 |
| deferoxamine CTD 00005759 | 2.87200421507584E-5 | CCNB1;VCAM1;FABP4;ADM |
| troglitazone CTD 00002415 | 3.1309270002551195E-5 | TOP2A;CCNB1;FABP4;ADM;HIST1H2BD;BCL2L1;FBN1 |
| tetracycline CTD 00006851 | 3.989655738992562E-5 | TOP2A;PLA2G7;BCL2L1 |
| oxytetracycline BOSS | 3.989655738992562E-5 | PLA2G7;BCL2L1;FBN1 |

Table S6. Details of gene-disease relationship.

| Term | P-values | Genes |
| --- | --- | --- |
| Polycystic Ovary Syndrome | 7.894119283734905E-6 | TOP2A;CCNB1;VCAM1;FABP4;PRKAR2B;PLA2G7;DKK1 |
| Cardiovascular Diseases | 1.1407771809779249E-5 | VCAM1;VNN1;FABP4;GGH;ADM;PLA2G7;DKK1;FBN1 |
| Vascular Diseases | 2.0109270562646106E-5 | VCAM1;FABP4;ADM;PLA2G7;FSTL1;FBN1 |
| Malignant neoplasm of urinary bladder | 2.2440742385346128E-5 | TOP2A;ESM1;CCNB1;FABP4;ARHGAP18;GGH;ADM;BCL2L1;APOBEC3B |
| Pulmonary Hypertension | 6.401855206246285E-5 | VCAM1;ADM;PLA2G7;BCL2L1 |
| Asthma | 6.641919867031292E-5 | VCAM1;VNN1;FABP4;ADM;RNASE3;PLA2G7;FSTL1;DKK1;BCL2L1 |
| Malignant neoplasm of ovary | 6.871254508148056E-5 | TOP2A;CCNB1;VCAM1;FABP4;ADM;RNASE3;FSTL1;DKK1;BCL2L1;FBN1;APOBEC3B |
| Ovarian Carcinoma | 1.475577760496719E-4 | TOP2A;CCNB1;VCAM1;FABP4;ADM;RNASE3;FSTL1;DKK1;BCL2L1;FBN1;APOBEC3B |
| OVERLAP CONNECTIVE TISSUE DISEASE | 1.567819481163321E-4 | DKK1;FBN1 |
| Acute asthma | 1.567819481163321E-4 | VNN1;RNASE3 |
| Carcinogenesis | 1.6943359795049348E-4 | TOP2A;VCAM1;ARHGAP18;GGH;ADM;RNASE3;FSTL1;DKK1;ESM1;CCNB1;FABP4;PRKAR2B;BCL2L1;FBN1;APOBEC3B |
| Diabetes Mellitus | 1.9173049922697235E-4 | ESM1;VCAM1;FABP4;ADM;RNASE3;PLA2G7;DKK1;SLC27A2;BCL2L1 |
| Ischemic stroke | 2.3260653203341402E-4 | VCAM1;FABP4;ADM;PLA2G7;FBN1 |
| Congenital contractural arachnodactyly | 2.3674137138859382E-4 | CCNB1;PRKAR2B;PLA2G7;FBN1 |
| Conventional (Clear Cell) Renal Cell Carcinoma | 2.460625642280338E-4 | ESM1;VCAM1;ADM;DKK1;BCL2L1;APOBEC3B |
| Bladder Neoplasm | 2.4950919843372944E-4 | TOP2A;ESM1;CCNB1;FABP4;GGH;ADM;BCL2L1;APOBEC3B |
| Colorectal Cancer | 3.201240434686009E-4 | TOP2A;HIST1H2BM;VCAM1;GGH;ADM;RNASE3;FSTL1;DKK1;ESM1;CCNB1;VNN1;HIST1H1B;BCL2L1 |
| Congenital keratoglobus | 3.418661725917297E-4 | CHRDL1;FBN1 |
| Gastric ulcer | 3.4683996824770526E-4 | VCAM1;ADM;BCL2L1 |
| Myocardial Infarction | 3.675410220671791E-4 | VCAM1;FABP4;C7;ADM;PLA2G7;FSTL1;BCL2L1 |
